# Survival from cancer of the uterus in England and Wales up to 2001

**DOI:** 10.1038/sj.bjc.6604591

**Published:** 2008-09-23

**Authors:** N Cooper, M J Quinn, B Rachet, E Mitry, M P Coleman

**Affiliations:** 1 Social and Health Analysis and Reporting Division, Office for National Statistics (Room FG/114), 1 Myddelton Street, London EC1R 1UW, UK; 2 Cancer Research UK Cancer Survival Group, Non-Communicable Disease Epidemiology Unit, Department of Epidemiology and Population Health, London School of Hygiene and Tropical Medicine, Keppel Street, London WC1E 7HT, UK; 3 Département d'Hépatogastroentérologie et Oncologie Digestive, Centre Hospitalo-Universitaire Ambroise-Paré, 9 avenue Charles de Gaulle, F-92100 Boulogne, France

Approximately 200 000 women were diagnosed with cancer of the body of the uterus around the world each year at the turn of the century. More than two-thirds of this estimated total arise in developed countries, where age-standardised incidence is four times higher than in developing countries (Ferlay *et al*, 2004).

In the late 1990s, there were around 5300 new cases a year of uterine cancer in England and Wales. It is the fifth most common cancer in women, accounting for 3.5% of all cases. Age-standardised incidence remained stable during the 1970s and 1980s, but it has risen by 25% since 1990 (Parkin *et al*, 2001; Quinn *et al*, 2001). Annual incidence in five deprivation groups defined by the woman's small area of residence at diagnosis ranged between 14 and 17 per 100 000 per year in the late 1990s, but there is no simple gradient across the groups, and incidence rose at a similar rate in all socioeconomic groups (data not shown). Mortality from uterine cancer has been declining steadily in all age groups except the very elderly (Quinn *et al*, 2001).

Women whose uterus has been removed are no longer at risk, and retaining them in the population denominators when estimating incidence rates artificially reduces the estimates. The prevalence of hysterectomy should be borne in mind when interpreting incidence rates and trends. In 1995, 2.3 million women in England and Wales had had a hysterectomy, with a peak prevalence of 21% in the age group of 55–59 years (Redburn and Murphy, 2001), so the estimated 25% rise in overall incidence since 1990 may be an underestimate of the increase.

Diagnosis of endometrial cancer may arise from postmenopausal bleeding, or irregular or heavy perimenopausal bleeding. If detected early, both endometrial carcinoma and *in situ* malignancy, which carries a high risk of progression to invasive cancer, are mostly curable. The mainstay of treatment is total hysterectomy, with or without removal of the ovaries, with external beam radiotherapy in some cases. Well-differentiated tumours with minimal invasion may be treated by brachytherapy with intravaginal irradiation.

Endometrial cancer shares some epidemiological features with breast and ovarian cancers, such as a peak in incidence around the menopause, and risk factors such as early menarche, low parity and late menopause. The risk of uterine cancer is also increased among women treated with tamoxifen for breast cancer. The unusual pattern of human carcinogenicity of tamoxifen has been summarised as follows: ‘There is *sufficient evidence* in humans for the carcinogenicity of tamoxifen in increasing the risk for endometrial cancer and there is conclusive evidence that tamoxifen reduces the risk for contralateral breast cancer in women with a previous diagnosis of breast cancer’ (IARC, 1996).

The survival analyses reported here involve more than 53 000 women aged 15–99 years who were registered with uterine cancer in England and Wales during the 14-year period 1986–1999, 87% of those who were eligible. Approximately 2% of women were excluded because their vital status was unknown on 5 November 2002 when the data were extracted for analysis; 5% because they had previously had a primary malignancy of another organ at some time since 1971, and a further 6% because their duration of survival was zero or unknown. The proportion excluded for zero or unknown duration of survival was similar in all deprivation categories, and did not change during the 1990s.

Cancers of the uterus are not normally registered without sufficient information being available for the cancer registry to assign the anatomic location as either the cervix or corpus uteri, but approximately 6% of tumours diagnosed in England and Wales during 1986–1999 had been assigned a non-specific anatomic site code (ICD-9 179, ICD-10 C55: malignant neoplasm of uterus, part unspecified); these tumours were considered ineligible for analysis.

More than half the tumours (58%) were coded as adenocarcinoma, 15% as endometrioid carcinoma, and almost 3% as leiomyosarcoma; 12% were poorly specified carcinomas.

## Survival trends

**Figure 1:**
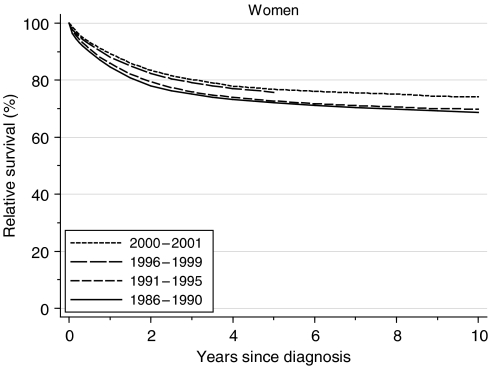
Relative survival (%) up to 10 years after diagnosis by calendar period of diagnosis: England and Wales, adults (15–99 years) diagnosed during 1986–1999 and followed up to 2001. Survival estimated with cohort or complete approach (1986–1990, 1991–1995, 1996–1999) or hybrid approach (2000–2001) (see Rachet *et al*, 2008).

Relative survival at both 1 and 5 years after diagnosis rose steadily and significantly from 85 and 72%, respectively, in the late 1980s, to 88 and 76% in the late 1990s. This represents a deprivation-adjusted rise of 2.5% every 5 years in short-term and longer-term survival (Table 1). The increase in 5-year survival was more marked between the early and late 1990s (Figure 1).

Short-term predictions suggest that these steady upward trends are likely to continue, with predicted survival of 90% at 1 year and 77% at 5 years, based on hybrid analysis (Brenner and Rachet, 2004) of survival probabilities observed during 2000–2001 (Table 1).

## Deprivation

The deprivation gap in survival between the most affluent and the most deprived groups is approximately 4%. This is a smaller gap in survival than for some other common cancers in women, but survival has nevertheless been consistently and significantly lower for women living in more deprived areas.

**Figure 2:**
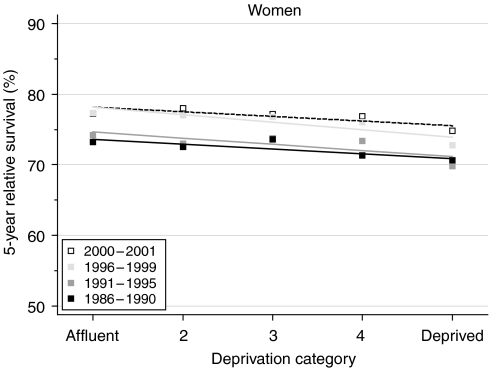
Trends in the deprivation gap in 5-year relative survival (%) by calendar period of diagnosis: England and Wales, adults (15--99 years) diagnosed during 1986--1999 and followed up to 2001.

The deprivation gap widened slightly but not significantly from approximately 3% for women diagnosed in the late 1980s to approximately 4% for those diagnosed in the late 1990s (Table 2 and Figure 2).

Predictions based on hybrid analysis of survival probabilities observed during 2000–2001 suggest that the deprivation gap for both short-term and longer-term survival is unlikely to change markedly within the next 5–10 years (Table 2).

## Comment

Survival from uterine cancer in England and Wales is amongst the highest for any cancer in women, after melanoma of the skin, and close to that for breast cancer. Survival has improved steadily in the 15 years up to 2001, and especially during the 1990s. Although survival has improved in all socioeconomic groups, the deprivation gap in survival between women in the most affluent and the most deprived groups has not narrowed; if anything, the data suggest that it widened slightly during the late 1990s.

Survival is closely related to stage at diagnosis, and the presenting symptom of postmenopausal bleeding in 90% of women leads to early diagnosis in the great majority. The very high survival in early-stage disease suggests that even greater attention could be given to encouraging early presentation, and to rapid referral, particularly for women in deprived socioeconomic groups.

In the early 1990s, relative survival from uterine cancer at 5 years ranged from 60 to 84% in the 22 European countries contributing to the EUROCARE-3 study. The age-adjusted European average 5-year survival of 76% was 2–3% higher than in England and 6% higher than in Wales (Sant *et al*, 2003).

## Figures and Tables

**Table 1:** Trends in relative survival (%) by time since diagnosis and calendar period of diagnosis: England and Wales, adults (15–99 years) diagnosed during 1986–1999 and followed up to 2001

		**Calendar period of diagnosis^a^**						**Average change (%)**		**Prediction^c^ for patients**	
		**1986–1990**		**1991–1995**		**1996–1999**		**every 5 years^b^**		**diagnosed during 2000–2001**	
**Time since diagnosis**		**Survival (%)**	**95% CI**	**Survival (%)**	**95% CI**	**Survival (%)**	**95% CI**	**Survival (%)**	**95% CI**	**Survival (%)**	**95% CI**
1 year	Women	**84.5**	(83.9, 85.1)	**85.9**	(85.3, 86.4)	**88.1**	(87.5, 88.6)	2.5**	(1.4, 3.6)	**89.3**	(88.5, 90.1)
5 years	Women	**72.0**	(71.2, 72.8)	**72.7**	(72.0, 73.5)	**75.8**	(74.8, 76.7)	2.5**	(0.9, 4.1)	**76.8**	(75.6, 77.9)
10 years	Women	**68.8**	(67.8, 69.7)	**69.9**	(68.9, 70.8)			**0.7**	(−2.7, 4.0)	**74.1**	(72.8, 75.4)

CI=confidence interval.

a Survival estimated with cohort or complete approach (see Rachet *et al*, 2008).

b Mean absolute change (%) in survival every 5 years, adjusted for deprivation (see Rachet *et al*, 2008).

c Survival estimated with hybrid approach (see Rachet *et al*, 2008).

^**^P<0.01.

**Table 2:** Trends in the deprivation gap in relative survival (%) by time since diagnosis and calendar period of diagnosis: England and Wales, adults (15–99 years) diagnosed during1986–1999 and followed up to 2001

		**Calendar period of diagnosis^a^**						**Average change (%)**		**Prediction^c^ for patients**	
		**1986–1990**		**1991–1995**		**1996–1999**		**every 5 years^b^**		**diagnosed during 2000–2001**	
**Time since diagnosis**		**Deprivation gap (%)**	**95% CI**	**Deprivation gap (%)**	**95% CI**	**Deprivation gap (%)**	**95% CI**	**Deprivation gap (%)**	**95% CI**	**Deprivation gap (%)**	**95% CI**
1 year	Women	−2.5**	(−4.2, −0.8)	**−1.3**	(−2.9, 0.3)	−3.9**	(−5.5, −2.3)	**−0.8**	(−2.0, 0.5)	−3.2**	(−5.4, −1.0)
5 years	Women	−2.7*	(−5.1, −0.4)	−3.5**	(−5.7, −1.2)	−4.2**	(−6.9, −1.6)	**−0.8**	(−2.7, 1.1)	**−2.6**	(−5.9, 0.6)
10 years	Women	−3.5**	(−6.2, −0.9)	−3.2*	(−6.0, −0.4)			**0.3**	(−3.5, 4.2)	**−2.5**	(−6.4, 1.4)

CI=confidence interval.

a Survival estimated with cohort or complete approach (see Rachet *et al*, 2008).

b Mean absolute change (%) in the deprivation gap in survival every 5 years, adjusted for the underlying trend in survival (see Rachet *et al*, 2008).

c Survival estimated with hybrid approach (see Rachet *et al*, 2008).

^*^P<0.05; ^**^P<0.01.
